# RDR1-mediated broad antitumor response: a novel strategy manipulating miRNAs as a powerful weapon

**DOI:** 10.1093/lifemedi/lnac007

**Published:** 2022-06-28

**Authors:** Ye Qi, Li Ding, Mingyi Xie, Peng Du

**Affiliations:** MOE Key Laboratory of Cell Proliferation and Differentiation, School of Life Sciences, Peking University, Beijing 100871, China; Peking-Tsinghua Center for Life Sciences, Academy for Advanced Interdisciplinary Studies, Peking University, Beijing 100871, China; MOE Key Laboratory of Cell Proliferation and Differentiation, School of Life Sciences, Peking University, Beijing 100871, China; Department of Biochemistry and Molecular Biology, University of Florida, College of Medicine, Gainesville, FL 32610, USA; UF Health Cancer Center, University of Florida, Gainesville, FL 32610, USA; UF Genetics Institute, University of Florida, Gainesville, FL 32610, USA; MOE Key Laboratory of Cell Proliferation and Differentiation, School of Life Sciences, Peking University, Beijing 100871, China; Peking-Tsinghua Center for Life Sciences, Academy for Advanced Interdisciplinary Studies, Peking University, Beijing 100871, China

Cancer is a group of diseases characterized by uncontrolled cell growth. The causes of cancer are very complex, including progressive accumulation of mutations, deregulation of key molecular pathways and of the microenvironment. Aberrant activation of the cell cycle is necessary for the abnormal proliferation of cancer cells. Therefore, cell cycle proteins are considered as attractive targets for drug development. However, existing drugs targeting one or more cell cycle proteins cannot overcome the drug resistance caused by the redundancy of cyclin and CDK gene families [1].

Interestingly, emerging evidence suggest that microRNAs (miRNAs) are important small RNA molecules closely related to the occurrence of various types of cancers. Many miRNAs can directly target multiple cell cycle genes, and a decreased expression of these miRNAs induces uncontrollable cell proliferation and tumorigenesis [2]. For miRNA biogenesis in mammals, primary miRNAs (pri-miRNAs) with stem-loop structures are cleaved by DROSHA/DGCR8 complex and DICER protein, which generates mature miRNA duplexes with 2-nt 3ʹ end overhang. These are recognized by and loaded into Argonaute (AGO) proteins to form functional RNA-induced silencing complexes (RISCs), which then regulate the expression of target genes [3]. miRNAs are indispensable for development and important for many physiological activities in normal tissues of mammals, whereas miRNA dysregulation or deficiency causes human diseases [4]. Global decrease of miRNA dosage has been reported in many human cancers, and artificial suppression of miRNA biogenesis is able to drive tumor formation [4]. However, potential universal features of miRNAs in primary tumors and cancer cells, which might be important to cause widespread miRNA deficiency, have not been characterized in detail till today.

In plants, RNA-dependent RNA polymerases (RDRs) are important immune proteins that specifically mediate siRNA production. Notably, RDR1 mediates an essential small RNA-dependent immune response against viral infection and spreading, which however is lost in vertebrates with adaptive immunity during evolution [5]. Since RDR is a key component that is absent in mammalian RNA silencing pathway and RDR1 mediates a plant-specific immune response, we propose to perform plant RDR1-based bioengineering in mammals and to further investigate its potential translational medicine applications [6].

To this end, RDR1 genes were cloned from *Arabidopsis thaliana* (At) and *Oryza sativa* (Os) into lentiviral vectors, used to infect mammalian cells and to obtain stable RDR1-inducible cell lines. Surprisingly, we observed repressed cell proliferation in all cancer cell lines tested, but not in all non-cancer cell lines. Both polyA(+) RNA-seq and EdU/PI staining assay showed that plant RDR1 can specifically target and interfere with the cell cycle process in cancer cells only. Next, we wanted to figure out the mechanism of the antitumor effect of plant RDR1. Although any kind of siRNAs could not be detected in cancer cells with RDR1 expression, we unexpectedly found a global increase of miRNA dosage in all cancer cell lines, but not in the non-cancer control cell lines. Since miRNAs can target multiple cell cycle genes, we next proved that RDR1 increased the global miRNA expression and inhibited the cell cycle process through the miRNA-mediated regulatory network.

To understand why and how RDR1 increases miRNA expression specifically in cancer cells, we performed isoform analysis on a large scale of small RNA-seq data from TCGA and GEO database. Surprisingly, we found that 1-nt-shorter miRNA isoforms, instead of the typical miRNAs derived from 2-nt-overhang duplexes, were widely accumulated in the cancer samples. Duplexes formed by these 1-nt-shorter miRNA isoforms were proved to be inefficiently loaded into AGO2 protein hampering the formation of functional RISCs, which might be related to the reduction of miRNA dosage in cancer cells. Since miRNA isoforms in cancer cells were not systematically characterized in the previous studies, our discovery provided a new perspective for studying problematic miRNA biogenesis or metabolism in cancer cells.

**Figure 1:**
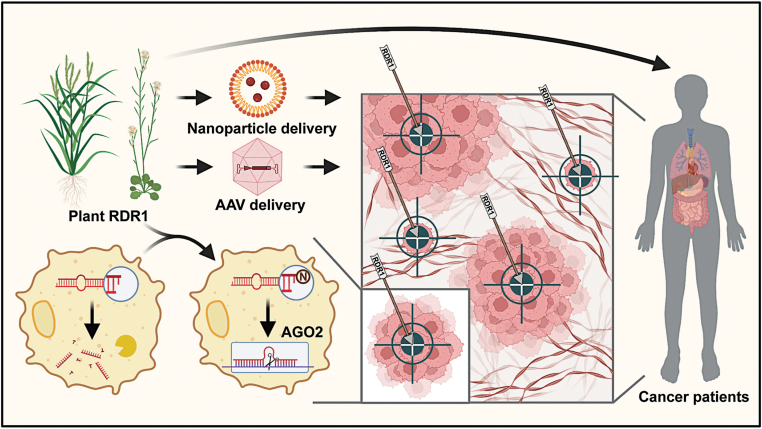
Illustration showing RDR1 enabling broad antitumor response by rescuing microRNA deficiency in cancers.

Considering that plant RDR6 has RNA polymerase and nucleotidyltransferase activities [7], we proposed that duplexes containing these AGO2-free 1-nt-shorter miRNA isoforms could be recognized and repaired by RDR1 through adding mononucleotide tails at the 3ʹ end. In the RDR1 tailing assay followed by miRNA/AGO2 loading assay, we observed that the loading efficiency of 1-nt-shorter miRNA duplexes was obviously increased after recovering their 2-nt overhang structure by mononucleotide tailing. Besides, by transfecting exogenous miRNA duplexes and tracking the proportion of these isoforms, we found that compared with 2-nt-overhang miRNA duplexes that can be efficiently loaded into AGO2, the 1-nt-overhang miRNA duplexes loaded into AGO2 were significantly reduced. These were more easily recognized and modified through mononucleotide tailing by RDR1, and these modified miRNAs then became the main population that were loaded into AGO2. To summarize, we propose that plant RDR1 with nucleotidyltransferase activity is able to add mononucleotide tails to the 1-nt-shorter miRNA duplex isoforms, which is dissociated from AGO2 and accumulated in cancer cells and enhances the AGO2 loading efficiency to finally rescue the defective miRNA pathway in cancer cells (Figure 1).

Surprisingly, antitumor effects of plant RDR1 were also observed in mice *in vivo*, showing broad potential for medical treatment using RDR1. We performed mouse xenograft experiments by injecting RDR1-inducible cancer cells into immunodeficient mice. In both solid tumor models and leukemia models, we found that the inducible expression of RDR1 inhibited tumor growth or prolonged life span of the xenotransplantation mice. Based on this result, we next aimed to test the possibility of RDR1 delivery to cancer cells. We observed that the proliferation of cancer cells was obviously suppressed together with a downregulation of cell-cycle gene expression after nanoparticle-mediated RDR1 delivery *in vitro* and AAV-mediated RDR1 delivery *in vivo* (Figure 1).

Although miRNAs have been studied for about 30 years, the distinct features of miRNA isoforms have not been systematically described in cancer. By large-scale data mining, we discovered that 1-nt-shorter miRNA isoforms were accumulated in cancer cells, which could not be loaded into AGO2 effectively. To figure out the potential key problematic step of miRNA biogenesis leading to the miRNA shortening in cancer cells, we checked published literature for potential mutations and analyzed the expressional changes of known essential components participating miRNA biogenesis, tailing or trimming based on published TCGA datasets. However, we did not find any common mutations or expressional changes of these known genes that are responsible for the global abnormal miRNA shortening in cancer cells, which is consistent with a recently published study [8]. Thus, we propose that there is another unknown mechanism controlling this cancer-specific 1-nt-shortening of miRNAs, which will be one of our research directions in the future.

Based on previous studies in plants, RDR protein owns both polymerase and nucleotidyltransferase enzymatic activities to synthesize dsRNAs and modify single-stranded RNAs, respectively [7]. Similarly, we also found that RDR1, by using its nucleotidyltransferase activity, can modify mammalian single-stranded miRNAs or double-stranded miRNA duplexes with 1-nt/2-nt overhangs, but not the duplexes with blunt ends. Very interestingly, *Neurospora crassa* QDE1, a homolog of RDR6, can also modify single-stranded RNA substrates with mononucleotides [9]. It is worthy to note that another nucleotidyltransferase TUT7 is also known to modify 1-nt-overhang pre-miR-let-7 with mono-uridylation, and TUT2 can add a single “A” to mature miRNAs [10]. Yet till today, it is still unclear why these nucleotidyltransferases, including some RDR proteins and TUTases, can only add mononucleotides and not long tails to single-stranded RNA substrates, which is of great interest and needs to be further explored.

Unlike vertebrates, plants do not have an adaptive immune system and mainly rely on molecular immunity. RNA silencing mediated by the immune protein RDR1 is one of the most important innate immunity approaches used by plants to counteract pathogens, including protists and viruses. During virus infection, RDR synthesizes viral replicative dsRNA intermediates which can be cleaved by host DICER and loaded into AGO to initiate the cleavage of cognate viral RNAs. Mechanically, RDR1-mediated viral silencing can ignore the occurrence of immune escape mutations in viral evolution. In future studies, we will further reconstitute this plant immune pathway in animal cells and investigate the potential antiviral activity of RDR1 protein in mammals.
